# BEHAVIORAL CHANGES OF NURSING STAFF AFTER A COACHING-BASED PSYCHOSOCIAL PROGRAM SUPPORTING SELF-DIRECTION IN PLWD

**DOI:** 10.1093/geroni/igad104.1120

**Published:** 2023-12-21

**Authors:** Maud Graff, Phebe Das, Gebrich Douma, Lieve Roets, Hanneke Donkers

**Affiliations:** Radboud University Medical Center, Nijmegen, Gelderland, Netherlands; Radboud University Medical Center, Nijmegen, Gelderland, Netherlands; Radboud University Medical Center, Nijmegen, Gelderland, Netherlands; Radboud University Medical Center, Nijmegen, Gelderland, Netherlands; Radboud University Medical Center, Nijmegen, Gelderland, Netherlands

## Abstract

The psychosocial person-centred intervention “SOCAV-in-primary-care” (SOCAV-PC) offers training and reflective peer-coaching to nursing staff and training and person-centred care-planning to caregivers. SOCAV-PC aims to support persons with dementia (PwD) to maintain/improve their self-direction and daily functioning in meaningful activities with support of their caregivers. This study presents the experiences of the PwD, and changes in attitudes and behaviour of caregivers and nursing staff when following SOCAV. Spread over nine months, caregivers and nursing staff were trained and coached in the person-centred approach by a trained SOCAV-peer coach. Content analysis was performed using qualitative data derived from (1) intervention and coaching diaries from nursing staff, (2) coaching diaries of peer coaches, (3) focus groups among nursing staff, and (4) individual interviews with PwD and their caregivers. PwD felt heard and respected in making their own choices. Caregivers became more aware of the influence of their own behaviour on the mood of the PwD, and started trying to convert their controlling behaviour, meant to provide safety, into supporting wishes and needs of PwD. Among nursing staff, an ongoing process of positive changes in attitudes and behaviours towards person-centred care emerged: from acting on intuition and automatisms, towards learning to reflect on the behaviour of PwD and their own actions. Moreover, they gained a better understanding of the concept of self-direction, and discovered the power of conversation and observation through a more open attitude and behavioural change. Reflective coaching is an essential tool to enhance person-centred attitudes and behaviour among nursing staff.

